# Amniotic Fluid Infection in Preterm Pregnancies with Intact Membranes

**DOI:** 10.1155/2017/8167276

**Published:** 2017-01-12

**Authors:** Tarja Myntti, Leena Rahkonen, Irmeli Nupponen, Anu Pätäri-Sampo, Minna Tikkanen, Timo Sorsa, Juuso Juhila, Sture Andersson, Jorma Paavonen, Vedran Stefanovic

**Affiliations:** ^1^Department of Obstetrics and Gynecology, Helsinki University Hospital and University of Helsinki, Helsinki, Finland; ^2^Children's Hospital, Helsinki University Hospital and University of Helsinki, Helsinki, Finland; ^3^Department of Bacteriology, University of Helsinki and Helsinki University Hospital, HUSLAB, Helsinki, Finland; ^4^Department of Oral and Maxillofacial Diseases, Helsinki University Hospital, Institute of Dentistry, University of Helsinki, Helsinki, Finland; ^5^Division of Periodontology, Department of Dental Medicine, Karolinska Institutet, Huddinge, Sweden; ^6^Medix Biochemica, Espoo, Finland

## Abstract

*Introduction*. Intra-amniotic infection (IAI) is a major cause of preterm labor and adverse neonatal outcome. We evaluated amniotic fluid (AF) proteolytic cascade forming biomarkers in relation to microbial invasion of the amniotic cavity (MIAC) and IAI in preterm pregnancies with intact membranes.* Material and Methods*. Amniocentesis was made to 73 women with singleton pregnancies; 27 with suspected IAI; and 46 controls. AF biomarkers were divided into three cascades: Cascade 1: matrix metalloproteinase-8 (MMP-8), MMP-9, myeloperoxidase (MPO), and interleukin-6; Cascade 2: neutrophil elastase (HNE), elafin, and MMP-9; Cascade 3: MMP-2, tissue inhibitor of matrix metalloproteinases-1 (TIMP-1), MMP-8/TIMP-1 molar ratio, and C-reactive protein (CRP). MMP-8 was measured by an immunoenzymometric assay and the others were measured by ELISA. Standard biochemical methods, molecular microbiology, and culture techniques were used.* Results*. MMP-8, MMP-9, MPO, elafin, and TIMP-1 concentrations were higher in IAI suspected cases compared to controls and also in IAI suspected cases with MIAC compared to those without MIAC when adjusted by gestational age at amniocentesis. All biomarkers except elafin and MMP-2 had the sensitivity of 100% with thresholds based on ROC-curve. Odd ratios of biomarkers for MIAC were 1.2-38 and 95% confidential intervals 1.0-353.6.* Conclusions*. Neutrophil based AF biomarkers were associated with IAI and MIAC.

## 1. Introduction

Intra-amniotic infection (IAI), defined as microbial invasion of the amniotic cavity (MIAC) with intra-amniotic inflammation, is common in women with preterm labor and exists with or without preterm prelabor rupture of membranes (PPROM) [1]. MIAC often leads to IAI [2]. However, intra-amniotic inflammation in the absence of MIAC also occurs [3]. Amniocentesis and amniotic fluid (AF) rapid biomarker testing may help in optimal timing of delivery.

Infection may induce the degranulation of interleukin- (IL-) 6 and matrix metalloproteinases (MMP), from polymorphonuclear neutrophils (PMNs) [4]. MMP-8 has been linked to MIAC [5–7], inflammation [8–10], histological chorioamnionitis (HCA) [11], and adverse neonatal outcome [12, 13]. Several AF biomarkers form proteolytic cascades. They are formed in inflammatory conditions after recruitment and activation of neutrophils, which release their subcellular granules, that is, degranulating tissue destructive enzymes (for example, MMP-8, MMP-9, and neutrophil elastase (HNE)). Those and other such enzymes can form cascades by activating each other. The process leads to degradation of extracellular matrix components and modulation of cytokines [4], which may lead in obstetrics to membrane rupture or ripening and softening of the cervix.

MPO is the activator of MMP-8 and MMP-9, while IL-6 can act as their inducer (Cascade 1). HNE is an activator of MMP-9, and elafin is an antiprotease of HNE [14]. Elafin is produced not only by neutrophils but also by epithelial cells [14]. Thus, HNE and MMP-9 form another PMN-derived proteolytic cascade (Cascade 2), to which elafin associates. The third cascade comprises MMP-2, tissue inhibitor of matrix metalloproteinases-1 (TIMP-1), MMP-8/TIMP-1 molar ratio, and C-reactive protein (CRP) (Cascade 3). MMP-2 and TIMP-1 are not produced by PMNs.

While many reports on AF biomarkers in PPROM pregnancies exist, such studies of MIAC and IAI in preterm pregnancies with presumed intact membranes are only few [3, 15, 16].

## 2. Materials and Methods

This prospective study was conducted at the Department of Obstetrics and Gynecology, University Hospital, Helsinki, Finland, between June 2013 and October 2015. The Institutional Review Board approved the study protocol (Ref. number 75/13/03/03/2013). A written informed consent was obtained from all patients.

### 2.1. Patients

The study population consisted of 73 women who underwent amniocentesis with singleton pregnancy and presumed intact membranes. Twenty-seven women had suspected IAI. Among those, the median gestational age at amniocentesis was 27^+4^ weeks, ranging from 22^+0^ to 31^+4^. IAI was suspected in the presence of preterm contractions with at least one of the following criteria: uterine tenderness, fetal tachycardia, infectious discharge from cervix, maternal plasma CRP > 10 mg/L, total blood white cell (WBC) count > 20 × 10^9^/L, or visible sludge at ultrasound examination. Forty-six pregnant women with no suspected IAI served as controls (Table 1). In the control group the indication for amniocentesis was mid-trimester chromosomal analysis in 24 cases (52%), evaluation of fetal lung maturity [*n* = 5 (11%)], or exclusion of fetal chronic hypoxia by erythropoietin measurement [*n* = 17 (37%)]. Among controls, the gestational age at amniocentesis was (median, range) 23^+1^ weeks (from 17^+0^ to 37^+5^). In the control group, five (11%) developed preeclampsia, four (9%) had insulin treated gestational diabetes, and four (9%) pregnancies were complicated by fetal growth restriction (fetal growth below -2 SD). Fetal structural anomaly, proven or suspected fetal aneuploidy, and diabetes type I were not eligible.

Gestational age was based on the first trimester ultrasonography screening. Membranes were presumed intact in the absence of any clinical signs of membrane rupture.

### 2.2. Collection of Samples and Laboratory Procedures

Transabdominal amniocentesis was performed by our study group members. AF samples were retrieved. MIAC was defined as a positive AF culture or positive bacterial PCR by 16S rRNA gene sequencing. The result of bacterial culture was available in 23 (85%) cases and in none of the controls and the result of AF-PCR in all cases and controls. The microbial analyses have been described in detail previously [15].

AF specimens were divided into aliquots, frozen and stored at −20°C until the biomarkers were analysed (Medix Biochemica, Espoo, Finland). AF-MMP-8 quantitation was made with a solid-phase immunoenzymometric assay (MMP-8 IEMA, Medix Biochemica, Espoo, Finland) and it was analysed according to the manufacturer's instructions. The absorbance measurement was done at 414 nm using a microplate reader (Multiskan, Thermo Fisher Scientific, Vantaa, Finland). Other biomarkers were analysed by using commercial enzyme-linked immunosorbent assay (ELISA): AF-elafin [Human Trappin-2 (elafin) ELISA kit, RayBiotech, Norcross, GA, USA], AF-HNE [polymorphonuclear (Human PMN Elastase) Sandwich ELISA kit, eBioscience, Vienna, Austria], AF-IL-6 [interleukin-6 (IL-6) ELISA kit, R&D Systems, Minneapolis, USA], AF-MPO [myeloperoxidase (MPO) ELISA kit, Immundiagnostik AG, Bensheim, Hesse, Germany], AF-MMP-2 [matrix metalloproteinase-2 (MMP-2) ELISA kit, R&D Systems, Minneapolis, USA], AF-MMP-9 [matrix metalloproteinase-9 (MMP-9) ELISA kit, R&D Systems, Minneapolis, USA], AF-CRP [human C-reactive protein (CRP) ELISA kit, R&D Systems, Minneapolis, USA], and AF-TIMP-1 [tissue inhibitor of metalloproteinases 1 (TIMP-1) ELISA kit, GE Healthcare, Buckinghamshire, UK]. The inter- and intra-assay coefficients of variation for elafin were <10% and <12%, respectively; for HNE < 5% and < 6%, respectively; for IL-6 < 5% and < 5%, respectively; for MPO < 3% and < 5%, respectively; for MMP-2 < 7% and < 13%, respectively; for MMP-8 < 6% and < 6%, respectively; for MMP-9 < 3% and < 8%, respectively; for CRP < 10% and < 10%, respectively; and for TIMP-1 < 12% and < 16%, respectively. The limit of detection for elafin was 0.005 ng/mL, for HNE 0.00198 ng/mL, for IL-6 0.0007 ng/mL, for MPO 0.294 ng/mL, for MMP-2 0.37 ng/mL, for MMP-8 0.4 ng/mL, for MMP-9 0.156 ng/mL, for CRP 0.010 ng/mL, and for TIMP-1 1.25 ng/mL. For AF-LD the intra-assay coefficient of variation (CV) was <2.3% and the limit of detection of the assay was 5 IU/L. For AF-Gluc the detection limit was 0.50 mmol/L. In the concentration levels less than 4 mmol/L the intra-assay CV was 4.7% and at concentration levels from 4.1 mmol/L to 42 mmol/L 1.9%.

Patients were managed according to our clinical practice guidelines. During the study period, only the results of AF-lactate dehydrogenase (LD), AF-glucose (Gluc), AF-PCR, and AF bacterial culture were available to obstetricians in charge. The methods of AF-LD and AF-Gluc testing have been described previously [15, 17]. If AF-LD was less than 419 IU/L and AF-Gluc was more than 0.7 mmol/L [15] in the absence of MIAC, pregnancy could continue and patients were followed up as outpatients.

MMP-8 belongs to proinflammatory neutrophil based cascade but represents also a known biomarker in this study. The cut-off value for AF-MMP-8 was 41.5 ng/mL, which has shown a sensitivity of 100%, specificity of 69%, PPV of 62%, and NPV of 100% for MIAC in a previous study [18] and was considered consistent with inflammation. IAI was determined as AF-MMP-8 > 41.5 ng/mL in the presence of MIAC.

### 2.3. Neonatal Outcome

Data on deliveries (spontaneous, induced, or caesarean section) and neonates were collected from the hospital records. The short-term outcome variables for neonates were birth weight, Apgar score at 1 min and 5 min of age, umbilical artery pH and base excess (BE), need for neonatal intensive care unit, need for a respirator, and the presence of clinical sepsis or blood culture positive sepsis. CRP and WBC levels were recorded.

The diagnosis of neonatal sepsis was set by a neonatologist. Neonatal sepsis was categorized into clinical sepsis and blood culture positive sepsis. Clinical sepsis was defined as blood culture negative infection with symptoms consistent with sepsis, abnormal blood values, and positive response to a minimum of 5-day antibiotic treatment. Abnormal blood values supporting clinical sepsis diagnosis included elevated CRP more than 20 mg/L, leukocytosis or leukopenia, increased neutrophil precursors, thrombocytopenia, and no signs of other infections. The symptoms of clinical sepsis were respiratory distress, apnoea, tachycardia, poor perfusion, low blood pressure, fever, hypoglycaemia, hyperglycaemia, irritability, feeding problems, lethargy, and convulsions. Sepsis was categorized as present or absent. All neonates with clinical or blood culture positive sepsis received antibiotics.

Other outcome measures for neonates were respiratory distress syndrome (RDS), bronchopulmonary dysplasia (BPD), necrotising enterocolitis (NEC), retinopathy of prematurity (ROP), intraventricular hemorrhage (IVH), periventricular leukomalacia (PVL), or death.

### 2.4. Statistical Analysis

All calculations were carried out using Microsoft Statistical Package for Social Sciences (SPSS Inc., Chicago, IL, USA) for Windows v22.0. Categorical variables were compared by the chi-square test or Fisher's exact test if the number of cases was under five. Data with continuous variables did not follow a normal distribution and were compared by Mann–Whitney *U* test. Amniotic fluid biomarkers were regressed on gestational age at sample collection and on gestational age at delivery, and adjusted *p* values were computed using the residual as a dependent variable. The correlations of biomarker concentrations with MMP-8 were tested with Spearman bivariate correlation. The receiver operating characteristics (ROC) curve was constructed, area under the curve (AUC) was estimated, and the sensitivity and specificity were calculated. Two-tailed tests were used. A *p* value less than 0.05 was considered significant.

## 3. Results

The selected characteristics of the study population are shown in Table 1. MIAC was found in 7 women (26%) with suspected IAI (cases) and in none of the controls. Candida species were detected in two cases; other microbes (six different) were detected in one case each. MIAC was polymicrobial in one case (14%).

### 3.1. AF Biomarkers

*Cascade 1*. As shown in Table 2, the median concentrations of all biomarkers were higher in IAI suspected cases than in controls. When adjusted by gestational age at amniocentesis, all biomarkers except AF-IL-6 were higher in IAI suspected cases than in controls (Table 2). Among IAI suspected cases, all biomarkers were associated with MIAC, also when adjusted by gestational age (Table 3). The accuracies and the most optimal cut-off values based on ROC-curve are shown in Table 4. Among Cascade 1 biomarkers, MMP-9 had the highest risk for MIAC, OR 4.5 (95% CI 1.3–15.3) (Table 4). 

*Cascade 2*. The median concentration of HNE did not differ between cases and controls, but the median concentration of elafin and MMP-9 was higher in cases than in controls, also when adjusted by gestational age (Table 2). All biomarkers in Cascade 2 were associated with MIAC, also when adjusted by gestational age (Table 3). The accuracies and the most optimal cut-off values based on ROC-curve are shown in Table 4. 

*Cascade 3*. The median concentrations of all biomarkers except AF-CRP were higher in cases than in controls (Table 2). When adjusted for gestational age only MMP-2 and TIMP-1 concentrations differed between cases and controls (Table 2). TIMP-1 and MMP-8/TIMP-1 molar ratio were associated with MIAC in IAI suspected cases. MMP-2 and CRP were not associated with MIAC, even when adjusted by gestational age (Table 3). The accuracies and the most optimal cut-off values based on ROC-curve are shown in Table 4. 

**Figure 1:**
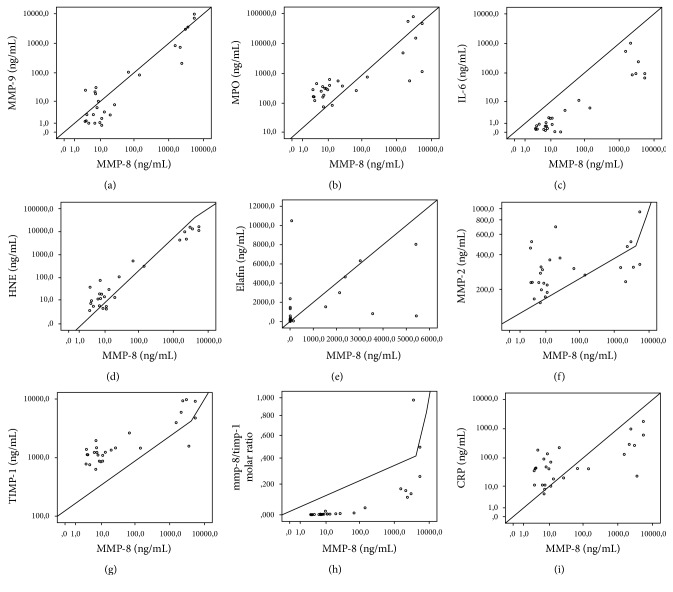
Correlation of other AF biomarkers with AF-MMP-8 among IAI suspected cases. Correlation values were (a) MMP-9 (*r*_*s*_ = 0.71, *p* < 0.001), (b) MPO (*r*_*s*_ = 0.748, *p* < 0.001), (c) IL-6 (*r*_*s*_ = 0.749, *p* < 0.001), (d) HNE (*r*_*s*_ = 0.749, *p* < 0.001), (e) elafin (*r*_*s*_ = 0.403, *p* = 0.037), (f) MMP-2 (*r*_*s*_ = 0.401, *p* = 0.038), (g) TIMP-1 (*r*_*s*_ = 0.717, *p* < 0.001), (h) MMP-8/TIMP-1 molar ratio (*r*_*s*_ = 0.949, *p* < 0.001), and (i) CRP (*r*_*s*_ = 0.501, *p* = 0.008).

**Figure 2:**
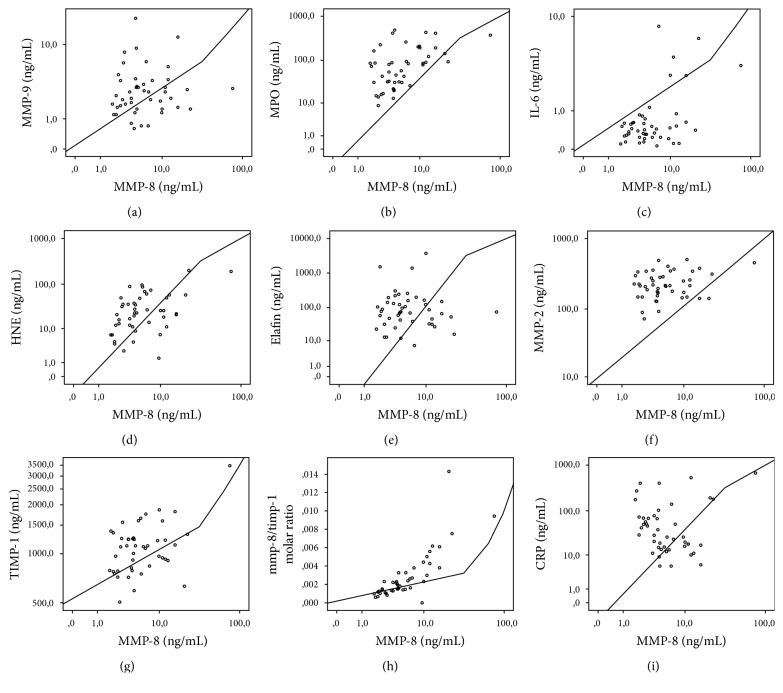
Correlation of other AF biomarkers with AF-MMP-8 among controls. Correlation values were (a) MMP-9 (*r*_*s*_ = −0.223, *p* = 0.21), (b) MPO (*r*_*s*_ = 0.609, *p* = 0.002), (c) IL-6 (*r*_*s*_ = 0.193, *p* = 0.367), (d) HNE (*r*_*s*_ = 0.671, *p* < 0.001), (e) elafin (*r*_*s*_ = 0.147, *p* = 0.49), (f) MMP-2 (*r*_*s*_ = 0.266, *p* = 0.21), (g) TIMP-1 (*r*_*s*_ = 0.179, *p* = 0.403), (h) MMP-8/TIMP-1 molar ratio (*r*_*s*_ = 0.845, *p* < 0.001), and (i) CRP (*r*_*s*_ = −0.294, *p* = 0.16).

Among IAI suspected cases, AF-HNE had the highest correlation with AF-MMP-8 (*r*_*s*_ = 0.749, *p* < 0.001), while AF-MMP-2 had the lowest one (*r*_*s*_ = 0.401, *p* = 0.038). Among controls AF-HNE had the highest correlation with AF-MMP-8 (*r*_*s*_ = 0.671, *p* < 0.001). The correlations of biomarkers except MMP-8 with MMP-8 separately in IAI suspected cases and in controls are shown in Figures 1 and 2.

In IAI cases (MMP-8 > 41.5 ng/mL with MIAC) AF-CRP correlated with MMP-8 (*r*_*s*_ = 0.501, *p* = 0.008), MMP-9 (*r*_*s*_ = 0.493, *p* = 0.044), MPO (*r*_*s*_ = 0.645, *p* < 0.001), IL-6 (*r*_*s*_ = 0.473, *p* = 0.013), and MMP-8/TIMP-1 molar ratio (*r*_*s*_ = 0.556, *p* = 0.003).

All biomarkers included were associated with IAI (*p* < 0.05) and all except MMP-2 (*p* = 0.053) were associated with AF inflammation (*p* < 0.002) (data not shown).

### 3.2. Neonatal Outcome

Of all IAI suspected cases, 11 neonates (41%) were born before 32 gestational weeks. Of these 11, six were born spontaneously within 7 days from amniocentesis (Table 5). Four of these were born after induction of labor due to suspected infection and one was born with caesarean section due to massive maternal hemorrhage. Nine neonates (33%) exhibited prematurity-related adverse neonatal outcome, all born ≤ 29^+0^ gestational weeks. Six (67%) of those had MIAC. Two neonates had early-onset neonatal sepsis.

Only one of the 16 neonates born after 32 gestational weeks was born from IAI-case where Ureaplasma was detected by AF-PCR. AF-LD and AF-Gluc were normal in this case suggesting colonization. AF-IL-6 concentration was 5.6 ng/mL and AF-MMP-8 concentration was 144 ng/mL, but these values were not available for the clinician. AF-MMP-9 (*p* = 0.006), MPO (*p* = 0.011), HNE (*p* = 0.034), and MMP-8/TIMP-1 molar ratio (*p* = 0.034) were associated with blood culture positive neonatal sepsis when the data were adjusted by gestational age at delivery (data not shown).

## 4. Discussion

We demonstrated that, among IAI suspected cases, all AF biomarkers from Cascades 1 and 2 were associated with MIAC, which was documented in one-fourth of our IAI suspected cases despite presumed intact membranes. The association persisted even after adjusting by gestational age at amniocentesis. Furthermore, in IAI cases AF biomarkers produced by neutrophils were associated with general inflammatory biomarker CRP [19] in AF. Additionally, neutrophil based biomarkers were able to both discriminate cases from controls after adjusting by gestational age at amniocentesis and associate with MIAC.

The problem with biomarkers commonly used, that is, AF-Gluc and AF-LD, is the limited accuracy for MIAC [15, 20, 21]. AF-MMP-8 and AF-IL-6 have been demonstrated to provide better accuracy [22] but are not commonly used in clinical practice. Rapid AF biomarkers are needed for optimal timing of delivery in women with suspected IAI.

We studied several AF biomarkers which form inflammation related proteolytic cascades. One can identify increased levels of PMN-derived biomarkers in MIAC cases. IL-6 can induce PMN extravasation at the site of inflammation, often triggered by microorganisms or virulence factors associated [4], and also act as a degranulation inducer.

Proinflammatory neutrophil-derived AF biomarkers (Cascade 1) associated with general inflammatory biomarker CRP in IAI cases though suggesting that activated and degranulating neutrophils are the major source of MMP-8 and MMP-9, MPO (Cascade 1), and HNE (Cascade 2) in the AF affected by MIAC. AF neutrophils are of maternal origin [1] reflecting maternal host response to infection. Cascade 3 biomarkers did not detect this since these biomarkers, MMP-2 and TIMP-1, are not produced and released by neutrophils [4, 23]. Furthermore, HNE and MPO can proteolytically and oxidatively inactivate TIMP-1, respectively, thus reducing the antiproteolytic defensive shield in AF [4, 23]. Infection leading to imbalance of MMP-8 and TIMP-1 may be related to initiation of preterm delivery [24].

MMP-9, IL-6, HNE, and elafin have been linked to MIAC in previous studies [14, 25–28], whereas MMP-2 has been associated with MIAC in PPROM pregnancies only [25]. TIMP-1 in AF has been linked to MMP-9 [29] and to MIAC in pregnancies with intact membranes [30], but in this study MIAC was not determined with PCR, as it was in our study. Thus, we confirmed and expanded the previous findings in IAI suspected cases with presumed intact membranes.

Several reports of AF-CRP and preterm delivery exist [31, 32], but only few include AF-CRP in the diagnosis of IAI [33]. In the study of Dulay exact AF-CRP values were not written out, but AF-CRP values differed by MIAC [33]. In that study both pregnancies with and without PPROM were included. Although AF-CRP is of fetal origin [34], we found that AF-CRP was not useful in IAI diagnostics or in predicting adverse neonatal outcome in pregnancies with presumed intact membranes. MIAC and funisitis have been associated with umbilical cord CRP-value [35], but our study had no intention to analyze postnatal cord samples.

We found that infection and inflammation occurred mainly at low gestational weeks, as also reported by Combs et al. [16]. So far, no randomized trials exist about the impact of amniocentesis in the management of preterm labor and PPROM on neonatal outcome. However, recent observational studies [36–39] suggest that it could be beneficial. Combs et al. [16] reported that AF infection or inflammation is associated with neonatal morbidity. However, we could not repeat this with any of the biomarkers studied. Smaller sample size and different clinical setting may explain this discrepancy.

Although this study had a relatively small sample size, we were able to show significant differences in biomarker concentrations between cases and controls. Multicentre study design is needed for larger sample size. Small sample size made multivariate analyses or adjustment of neonatal outcomes by gestational age meaningless. Larger studies are necessary to determine biomarker cut-off values for clinical practice and to find out whether a biomarker panel would be better than any single biomarker. The results have not been adjusted for gestational diabetes and preeclampsia, which is a limitation since some controls did have those background diseases, which may have some immune contribution. However, in the clinical practice it is important to find a biomarker working in the whole population, not just in those without any background diseases. Another limitation is the lack of molecular biology techniques in the definition of neonatal sepsis and the lack of microbial culture analysis in controls in the assessment of MIAC.

To our knowledge, this is the first study reporting the association of inflammation related proteolytic biomarker cascades with IAI. In summary, our results highlight the association of PMN-derived biomarkers with MIAC and IAI in preterm pregnancies with intact membranes.

## Figures and Tables

**Table 1:** Selected characteristics of the study population.

	Cases (*N* = 27)	Controls (*N* = 46)	*p* value
Maternal age, median (range)	31 (20–43)	32.5 (17–48)	0.23
Nulliparous, *n* (%)	10 (37)	21 (46)	0.47
BMI, median (range)^*∗*^	27 (20–49)	25.5 (18–48)	0.77
Smoking, *n* (%)	5 (18.5)	9 (20)	0.91
Gestational diabetes, *n* (%)	5 (18.5)	18 (39)	0.067
AC-delivery interval in weeks, median (range)	7.4 (0–17.7)	8.2 (0–24.3)	0.032
Gestational age at AC in weeks, median (range)	27.6 (22–32)	23.1 (17–38)	0.54
Gestational age at delivery in weeks, median (range)	37.9 (24–42)	38.3 (28–42)	0.013

^*∗*^Missing  *n* = 1.

BMI, body mass index; AC, amniocentesis.

**Table 2:** Amniotic fluid biomarker concentrations in IAI suspected cases and controls.

Biomarker ng/mL	Cases (*N* = 27)	Controls (*N* = 46)	*p* value	Adjusted *p*value^*∗*^
*Cascade 1*				
MMP-8	11 (3–5431)	4.5 (2–76)	<0.001	0.004
MMP-9	10 (0.7–9678)	2.6 (0.6–19)	0.003	0.005
MPO	362 (75.6–79095)	80 (8.6–488)	<0.001	<0.001
IL-6	1.1 (0.03–1021)	0.4 (0.06–8.2)	0.004	0.163
*Cascade 2*				
HNE	19 (2.8–16380)	23 (1.5–200)	0.51	0.444
Elafin	288 (7–10491)	74.5 (7–3739)	0.001	0.002
MMP-9	10 (0.7–9678)	2.6 (0.6–19)	0.003	0.005
*Cascade 3*				
MMP-2	296 (153–938)	216 (72–505)	0.01	0.01
TIMP-1	1347 (633–9752)	1112 (505–3473)	0.011	0.022
MMP-8 /TIMP-1 molar ratio	0.005 (0.001–0.973)	0.002 (0–0.014)	<0.001	0.069
CRP	43 (5–1783)	28.8 (5–670)	0.343	0.424
*Biomarkers routinely used in clinical practice*				
LD IU/L°	234 (88–5459)	14.5 (4.0–156)	<0.001	
Gluc mmol/L°	1.9 (0.0–4.0)	1.7 (0.3–4.9)	0.585	

Median (range).

°Missing *n* = 2 in controls.

^*∗*^Adjusted by gestational age at amniocentesis.

MIAC, microbial invasion of the amniotic cavity.

MMP-8, matrix metalloproteinase-8; MMP-9, matrix metalloproteinase-9; MPO, myeloperoxidase; IL-6, interleukin-6; MMP-2, matrix metalloproteinase-2; HNE, neutrophil elastase; TIMP-1, tissue inhibitor of matrix metalloproteinase-1; CRP, C-reactive protein; LD, lactate dehydrogenase; Gluc, glucose.

**Table 3:** Amniotic fluid biomarker concentrations in IAI suspected cases with or without MIAC.

Biomarker ng/mL	MIAC+ (*N* = 7)	MIAC− (*N* = 20)	*p* value	Adjusted *p*value^*∗*^
*Cascade 1*				
MMP-8	3019 (69–5431)	7.5 (3–2372)	<0.001	<0.001
MMP-9	2981 (84–9678)	3.4 (0.7–730)	<0.001	<0.001
MPO	4866 (267–79095)	299 (75.6–55185)	0.002	0.001
IL-6	94.9 (5.6–540)	0.6 (0.03–1021)	<0.001	0.002
*Cascade 2*				
HNE	11280 (315–16380)	12.3 (2.8–9862)	<0.001	<0.001
Elafin	1533 (58–10491)	191 (7–4642)	0.031	0.016
MMP-9	2981 (84–9678)	3.4 (0.7–730)	<0.001	<0.001
*Cascade 3*				
MMP-2	311 (266–938)	231.5 (153–696)	0.081	0.055
TIMP-1	3958 (1456–9752)	1178 (633–9317)	0.001	0.001
MMP-8 /TIMP-1 molar ratio	0.167 (0.011–0.973)	0.004 (0.001–0.155)	<0.001	<0.001
CRP	133 (23–1783)	42.5 (5–985)	0.092	0.092
*Biomarkers routinely used in clinical practice*				
LD IU/L	1689 (337–5459)	185.5 (88–1521)	<0.001	
Gluc mmol/L	0.1 (0.0–1.5)	2.0 (0.0–4.0)	<0.001	

Median (range).

^*∗*^Adjusted by gestational age at amniocentesis.

MIAC, microbial invasion of the amniotic cavity.

MMP-8, matrix metalloproteinase-8; MMP-9, matrix metalloproteinase-9; MPO, myeloperoxidase; IL-6, interleukin-6; HNE, neutrophil elastase; MMP-2, matrix metalloproteinase-2; TIMP-1, tissue inhibitor of matrix metalloproteinase-1; CRP, C-reactive protein; LD, lactate dehydrogenase; Gluc, glucose.

**Table 4:** The accuracies of biomarkers based on ROC-curve and the odd ratios OR (95% CI) for MIAC.

	Cut-off value ng/mL	AUC (95% CI)	Sensitivity	Specificity	OR (95% CI)
*Cascade 1*					
MMP-8	48	0.985 (0.96–1.0)	100	95.5	3.3 (1.3–8.6)
MMP-9	57.5	0.991 (0.97–1.0)	100	97	4.5 (1.3–15.3)
MPO	264	0.952 (0.88–1.0)	100	74.2	1.4 (1.1–1.8)
IL-6	5.1	0.974 (0.94–1.0)	100	93.9	2.8 (1.3–6.0)
*Cascade 2*					
HNE	257	0.987 (0.97–1.0)	100	97	4.5 (1.3–15.3)
Elafin	488.5	0.854 (0.68–1.0)	85.7	86.4	38 (4.1–353.6)
MMP-9	57.5	0.991 (0.97–1.0)	100	97	4.5 (1.3–15.3)
*Cascade 3*					
MMP-2	300.5	0.781 (0.65–0.91)	85.7	71.2	14.8 (1.7–131.7)
TIMP-1	1420.5	0.933 (0.87–1.0)	100	80.3	1.5 (1.1–2.1)
MMP-8 /TIMP-1 molar ratio	0.01	0.985 (0.96–1.0)	100	93.9	2.8 (1.3–6.0)
CRP	22	0.738 (0.56–0.91)	100	40.9	1.2 (1.0–1.3)

MIAC, microbial invasion of the amniotic cavity.

MMP-8, matrix metalloproteinase-8; MMP-9, matrix metalloproteinase-9; MPO, myeloperoxidase; IL-6, interleukin-6; HNE, neutrophil elastase; MMP-2, matrix metalloproteinase-2; TIMP-1, tissue inhibitor of matrix metalloproteinase-1; CRP, C-reactive protein; LD, lactate dehydrogenase; Gluc, glucose.

**Table 5:** Inflammation and MIAC by gestational age at delivery among cases.

	<32 + 0 weeks	>32 weeks	*p* value
*n*	11	16	
Gestational age in weeks at AC	29 (27–31)	30 (22–31)	0.8
AF MMP-8 concentration at AC, ng/mL	2131 (3–5431)	7.1 (3.1–144)	0.019
AF inflammation, *n* (%)	8 (73%)	1 (6%)	0.031
AF MIAC, *n* (%)	6 (55%)	1 (6%)	0.10
Spontaneous preterm birth < 7d following AC, *n* (%)	6 (55%)	0	<0.001

Median (range).

Inflammation, MMP-8 > 41.5 ng/mL.

AF, amniotic fluid.

AC, amniocentesis.

MIAC, microbial invasion of the amniotic cavity.

MMP-8, matrix metalloproteinase -8.
